# MCM immunocytochemistry as a first line cervical screening test in developing countries: a prospective cohort study in a regional cancer centre in India

**DOI:** 10.1038/sj.bjc.6603679

**Published:** 2007-03-06

**Authors:** G Mukherjee, B Muralidhar, U D Bafna, R A Laskey, N Coleman

**Affiliations:** 1 Department of Pathology, Kidwai Memorial Hospital Institute of Oncology, Bangalore, India; 2 Medical Research Council Cancer Cell Unit, Cambridge, CB2 2XZ, UK; 3 Department of Gynaecology, Kidwai Memorial Hospital Institute of Oncology, Bangalore, India

**Keywords:** cervix, screening, diagnostic accuracy, minichromosome maintenance proteins, immunocytochemistry

## Abstract

Cervical screening is not available for the majority of women in resource-poor countries. An important factor is a lack of skilled operators necessary for high-throughput assessment of the Papanicolaou (Pap) test currently in use. We compared the efficacy of immunocytochemistry for minichromosome maintenance (MCM) proteins *vs* standard Pap testing at detecting disease in 455 cervical smears processed in a typical Indian screening laboratory. Conventional (non-monolayer) smears were stained manually and then examined by a cytotechnologist and a cytopathologist. The MCM test was called positive when immunolabelled cells were identified as dyskaryotic by the Pap counterstain. The MCM test was read more quickly than the Pap test (approximately 2 *vs* 10 min) and there was 100% inter-observer agreement compared with 85% for Pap (*P*<0.0001). The MCM test detected 10 biopsy-proven cancers or pre-cancers that were not detected by Pap (*P*=0.002; *P*=0.016 excluding three cases where the Pap was deemed unsatisfactory on review). The cases in question included one recurrent squamous carcinoma and one adenocarcinoma in a screening patient who would have returned to 5 year recall. There were no false positive MCM test results. We propose that MCM immunocytochemistry has considerable advantages for cervical screening in developing countries like India.

Carcinoma of the uterine cervix is the most common malignancy in women in India, with over 1 26 000 cases per year (Nandakumar, 1996). More than 80% of these are diagnosed at an advanced stage, leading to 5 year survival rates of <40% (Shankarnarayanan, 1998; Dinshaw *et al*, 1999). The majority of cases represent squamous cell carcinoma (SCC), although adenocarcinoma and adenosquamous carcinoma are also encountered. Cervical screening in India is currently restricted to specialist institutes only and is therefore not available for the vast majority of the population. Widespread adoption of a practicable screening test could potentially decrease disease incidence and improve patient survival, as has been accomplished in developed nations (Baldwin *et al*, 2003).

For a cervical screening test to be adopted, the combined cost of performing and reading it must be affordable. The current Papanicolaou (Pap) test is difficult to assess, particularly for the conventional smears used in India, where liquid based cytology (LBC) is not affordable. The Pap test requires skilled operators, who are expensive to educate and retain in post. This leads either to the test not being adopted or, at the best, to it being performed infrequently, rather than regularly as part of a cervical screening programme. In this context, the widely acknowledged limitations on sensitivity and specificity of a single Pap test would substantially limit clinical effectiveness (Baldwin *et al*, 2003). A screening test that can be assessed quickly and/or by less highly trained operators, while remaining effective in detecting disease, would potentially be affordable in resource-limited countries. We have tested the value in such a setting of immunocytochemical staining for minichromosome maintenance (MCM) proteins 2–7, as biomarkers of abnormal cervical cells.

MCM proteins 2–7 are essential for DNA replication in all eukaryotic cells and for restricting replication to once per cell cycle (Kearsey and Labib, 1998; Gonzalez *et al*, 2005). These proteins, which are abundant throughout the cell cycle (Kearsey *et al*, 1996; Maiorano *et al*, 1996), are downregulated following cell cycle exit by quiescence, differentiation or senescence (Musahl *et al*, 1998; Madine *et al*, 2000; Stoeber *et al*, 2001). In normal cervical epithelium, MCMs are confined to the basal proliferative compartment and are absent from terminally differentiated superficial keratinocytes (Williams *et al*, 1998; Freeman *et al*, 1999; Baldwin *et al*, 2003). In contrast, in pre-malignant cervical intraepithelial neoplasia (CIN), the cellular proliferative compartment expands in proportion to histological grade, resulting in MCM-positive cells being present at the epithelial surface. We demonstrated that such aberrant expression can be exploited to improve detection of abnormal cells in cervical smears (Williams *et al*, 1998) and subsequently showed that the principle can be extended to early detection of neoplasia at other sites, including large bowel (Davies *et al*, 2002), larynx (Chatrath *et al*, 2003) and oral cavity (Scott *et al*, 2006).

In the present study, we have conducted an initial investigation of the suitability of MCM staining for the detection of abnormal cells in cervical smear samples from 455 Indian women. Our objective was to compare the efficacy of immunocytochemistry for MCMs (using pooled antibodies against MCM2 and MCM5) *vs* standard Pap testing, in conditions that apply in typical Indian cervical screening laboratories. To this end, conventional (i.e. non-monolayer) slides underwent manual staining and were examined by a cytotechnician and subsequently by a consultant cytopathologist. Our data will inform the design of appropriately powered large-scale future studies.

## MATERIALS AND METHODS

### Smear samples

**Figure 1:**
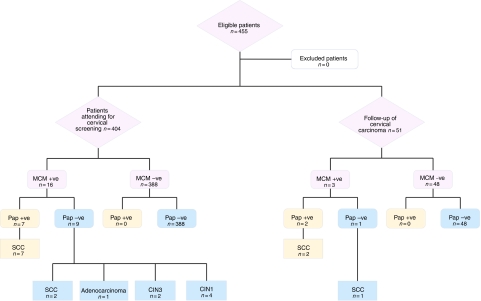
Flow chart summarising study design and results. ^*^For three cases in this group, the Pap smear was deemed unsatisfactory, rather than negative, on review.

In a prospective study, samples were obtained from 455 women who attended the gynaecology clinic at Kidwai Cancer Hospital in Bangalore, India between January and August 2004 (Figure 1). The study was approved by the Kidwai Local Research Ethics Committee (reference: PER/CAB-I/D-I/13/01). The sample population included 404 non-selected women who voluntarily attended for cervical screening examination (available for a small fee in Kidwai Memorial Cancer Hospital) and 51 consecutive women undergoing follow-up for treated cervical carcinoma. All subjects invited to take part in the study agreed to do so. Two samples were collected from each individual by a specialist gynaecologist, using a cervical cytobrush (TriPath, Burlington, NC, USA). The tip of the cytobrush was placed at the cervical os and rotated gently through 360° three times to obtain each sample. There were no adverse events in any of the subjects. Smears were prepared by spreading the samples uniformly across glass slides, which were immediately cytofixed (Surgipath, Richmond, IL, USA). The first smear was used for conventional Pap staining, which, as the standard cervical screening method in India, served as the reference test for the study. The second smear was used for immunocytochemistry, employing mouse monoclonal antibodies against human MCM2 and MCM5 (Scott *et al*, 2006). The tests were performed and assessed within 24 h of each other. Cases were managed clinically according to test results (see below). Biopsies were taken if abnormalities suggestive of cervical malignancy or pre-malignancy were visualised, either at the time of initial specialist examination or during any subsequent colposcopy.

### Immunohistochemistry

Immunostaining was carried out as described previously (Williams *et al*, 1998). In brief, smears were soaked in methanol for 5 min to remove cytofix. Cells were then permeabilised with 4 mM sodium deoxycholate for 10 min, washed in Tris-buffered saline (TBS) containing 0.25% Triton X-100, and blocked overnight with 10% goat serum in TBS. Pooled primary antibodies were diluted in TBS containing 1% bovine serum albumin (anti-MCM2 at 1 : 50 and anti-MCM5 at 1 : 20) and 200 *μ*l was added to each smear. Slides were incubated overnight at 4°C in a humidified chamber on an orbital shaker, then washed in TBS and incubated for 1 h at room temperature with biotinylated goat anti-mouse secondary antibody (DAKO, Ely, UK) at 1 : 200. Following quenching of endogenous peroxidase activity in 0.6% hydrogen peroxide for 5 min, signal was detected using streptavidin horseradish peroxidase and diaminobenzidine (both DAKO, Ely, UK). The reaction was stopped by rinsing in water and slides were counterstained with the standard Pap method (to produce an ‘immunoenhanced’ Pap stain). Negative controls were performed by omitting the primary antibodies. Tissue sections showing CIN were used as positive controls (Freeman *et al*, 1999).

### Smear evaluation and statistical analysis

Both the Pap and the MCM stained slides were examined by a cytotechnician and then by one of two consultant cytopathologists, who were blinded to the opinion of the cytotechnician, in order to test inter-observer variation. When assessing the Pap test, the observers were blinded to the results of the MCM test and vice versa. All the observers had over 15 years experience in their respective posts. All available clinical information was provided to each observer. Any discrepant diagnoses were subsequently resolved by consensus between all three observers.

The Pap test was reported using ABC2 criteria (Johnson and Patnick, 2000). The test was regarded as positive if there was a diagnosis of mild dyskaryosis or worse, as the former is the trigger point for further clinical intervention in Kidwai Memorial Hospital. Borderline changes in squamous or glandular cells were considered to represent negative results. The MCM test was regarded as positive if there was nuclear staining in epithelial cells in which features of mild dyskaryosis or worse could be identified from the Pap counterstain.

Further patient management depended on the results of the MCM and Pap tests. Cases that were positive by either test were reviewed and patients underwent colposcopy and biopsy (see Results section for details). In total, 19 of the 455 patients in the study underwent biopsy. All biopsies were reported using the CIN classification by a consultant histopathologist, with over 15 years experience, who was blinded to the results of the cytological tests.

The results of the MCM test were compared with the findings in the accompanying Pap smear and with the histopathological diagnosis in tissue samples (where available). The non-parametric McNemar's test (two-sided) was used to determine whether one test was significantly more sensitive than the other at detecting cervical malignancy (of any type) and pre-malignancy (CIN of any grade and cervical glandular intraepithelial neoplasia), as well as to compare inter-observer variation in assessing the two tests.

## RESULTS

### Assessing MCM stained cervical smears

**Figure 2:**
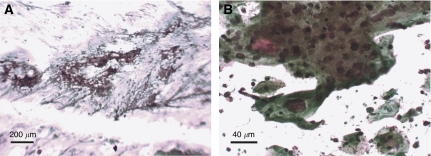
(**A** and **B**) Representative images of the immunoenhanced Pap test, showing MCM-positive cells at (**A**) low power and (**B**) high power.

The MCM test was evaluated in the setting of a typical Indian cervical screening laboratory. Manually stained cervical smears were examined by a cytotechnician and one of two consultant cytopathologists. In all cases where immunopositive cells were identified, they were seen readily, even at low magnification (Figure 2A). The Pap counterstain enabled the features of the immunopositive cells to be examined by the observers (who, it should be noted, were experienced in assessing such features) (Figure 2B). Of the 455 smears examined, 19 were MCM positive but identified as not showing mild dyskaryosis or worse (therefore being test negative) by the Pap counterstain.

Importantly, the time taken to read the MCM test slides was much shorter than for the Pap smears (approximately 2 min *vs* 10 min). Moreover, there was 100% agreement between observers in calling the MCM test in this study. In comparison, the inter-observer agreement for Pap stained slides was significantly lower at only 85% (387 of 455 smears) (*P*<0.0001; *n*=455; McNemar's Test).

### Patients attending for cervical screening

Between January and August 2004, samples were collected from 404 patients aged from 25 to 75 years, who had attended solely for cervical screening. Of the 404 cases, 388 were MCM negative and 16 were MCM positive. All 388 cases negative for MCM were reported as either inflammatory or negative in the accompanying routine Pap smears: no MCM negative case showed an abnormality by Pap testing. All 16 cases positive for MCM underwent further examination and biopsy. The findings were as follows:

- Three MCM positive cases were initially reported as negative by Pap smear. On review, it was decided that the quality of these smears was not satisfactory. This led to repeat smears, which in all three cases were reported as showing borderline squamous abnormalities. All three patients then underwent colposcopy and biopsy, from which two cases were diagnosed as CIN1 and one as CIN3.
- Three MCM positive cases were reported as borderline squamous abnormalities in the Pap smear. All three patients underwent colposcopy and biopsy, from which two cases were diagnosed as CIN1 and one as CIN3.
- One case was reported as showing borderline glandular abnormalities in the Pap smear. The patient was demonstrated to have adenocarcinoma of the cervix on biopsy.
- Two cases showed only necrosis on Pap smear. Both were confirmed as SCC on biopsy.
- Four cases were diagnosed as severe dyskaryosis by Pap smear and all proved to be SCC on biopsy.
- Three cases were diagnosed as showing SCC on Pap smear and all were confirmed by biopsy.

### Patients attending for follow-up of previously diagnosed cervical carcinoma

Fifty-one patients in the study were under routine follow-up, having previously received treatment (combinations of surgery/radiotherapy/chemotherapy) for cervical carcinoma. The patients were aged between 25 and 75 years. No patient was undergoing active therapy and none reported symptoms attributable to the cervix or lower genital tract. Of these samples, 48 were negative by MCM staining. None of these cases showed an abnormality by Pap testing. The three MCM-positive cases underwent further analysis as follows:

- One patient showed borderline squamous abnormalities on Pap staining. An indurated area of tissue was seen at colposcopy, which on biopsy was found to be SCC.
- Two patients showed SCC on Pap staining. In both cases, SCC was confirmed on biopsy.

### Comparison of MCM Testing *vs* Pap Testing

Overall, the combination of MCM staining and Pap counterstaining enabled the diagnosis of 10 cases of biopsy-proven cervical malignancy or pre-malignancy that were missed using Pap staining (*P*=0.002; *n*=455; McNemar's Test). Even excluding the three unsatisfactory Pap smears in the patients attending for routine screening (group (a) in the cervical screening section above), seven of the 455 smears examined were false negatives by Pap staining, with the sensitivity of MCM staining being significantly superior (*P*=0.016; *n*=452; McNemar's Test). There was no evidence of reduced specificity with the MCM test, which produced no false positive results. As the patients with Pap negative, MCM negative smears did not undergo further examination and biopsy, it was not possible to determine the absolute sensitivity, specificity and negative predictive value for either test. The positive predictive value for the MCM test in this initial study was 100% for CIN1 or worse (19/19 cases) and 79% (15/19 cases) for CIN3 or worse.

## DISCUSSION

The abnormal expression of MCMs in pre-malignant and malignant lesions of the cervix make them potentially excellent biomarkers for detecting abnormally proliferating cells (Gonzalez *et al*, 2005) and led us to develop a modification of the standard Pap smear that incorporated MCM immunocytochemistry (the immunoenhanced Pap test) (Williams *et al*, 1998). Pap staining, the traditional method used to detect abnormal cervical epithelial cells, is subjective and error-prone if conducted in isolation (Baldwin *et al*, 2003), as affordable screening would have to be in developing nations. Pap testing of LBC samples has been approved as a replacement for the conventional Pap smear and is considered to be significantly more effective at detecting cervical lesions (Kirschner *et al*, 2006). However, LBC is not being used in any of the major hospitals in India at present, for economic reasons. In view of the limitations of Pap testing in cervical smears, there remains a pressing need for alternative tests of greater efficacy. We therefore examined whether MCM staining would be a superior method of detecting cervical pre-cancer and cancer in the setting of a typical Indian screening laboratory, using conventional (non-monolayer) smears and without the deployment of additional facilities or technology.

Combined MCM staining and Pap counterstaining detected 10 cases of biopsy-proven cervical cancer or pre-cancer that were missed using Pap staining. In three of these cases, the Pap smears were reclassified as inadequate on review and had to be repeated. Interestingly, the MCM test was adequate for the identification of abnormal cells in the sample that accompanied the Pap stained slide in these three cases. As the Pap and MCM test slides were obtained separately, we cannot exclude that this difference was due to sampling bias. These cases not withstanding, the sensitivity of the MCM test was still significantly superior to Pap testing. Absolute sensitivity values cannot be given for either test as no tissue diagnosis was made on the Pap negative, MCM negative cases, for practical and financial reasons. Nevertheless, our findings have important clinical implications, as several women whose abnormalities were missed by Pap testing would not have undergone further investigation based on their Pap test result. The abnormalities in these women included one case of CIN3 and one adenocarcinoma in the cervical screening subjects and one SCC in the follow-up patients. For the screening group, the interval to the next clinical examination would have been at least 5 years.

The MCM test showed no reduction in specificity in our study, with no false positive results. It should be noted that the criteria used to diagnose MCM test positive cases required analysis by a consultant cytopathologist, assessing both staining results and the morphology of the immunopositive cells. MCMs can be expressed in cells showing reactive features (Williams *et al*, 1998), and whereas the morphology of reactive cells can generally be identified by experienced observers, any algorithms that did not take such information into account would generate reduced specificity for disease detection.

MCM testing led to a significant decrease in the inter-observer variation. This is attributable to the fact that all observers are able to assess the same cells, as they are readily located by virtue of their immunopositivity. Moreover, MCM testing substantially decreased the time needed to assess patient samples, an issue that is of great importance in the developing world due to the shortage of trained personnel. The MCM staining process would add cost to the conventional Pap test and require additional manual handling steps. However, the levels of skill and training required for this task are not unduly high and would be attainable and affordable. Such extra investment would be strongly counterbalanced by an important benefit of MCM testing, namely that test interpretation is less labour intensive and time consuming for the cytoscreeners and cytopathologists that are in such short supply. This fact, when viewed in combination with the improved test performance, argues that MCM staining may be of greater benefit and cost effectiveness than the Pap test as a primary screening method, including in developing countries like India.

An additional consideration is that, in the longer term, the analysis of MCM-stained slides has considerable potential for automation. This would further improve test throughput and would provide an economy of scale that would facilitate affordable screening in the large populations of developing countries. However, as such an approach would be best suited to LBC samples, the clinical and economic benefits of the various permutations of methods for obtaining and processing samples would need careful evaluation. It is also the case that the likelihood of successful adoption of a screening test depends on the availability of affordable methods to diagnose and treat the target disease effectively in the screened populations.

In summary, this study confirms that MCM immunocytochemistry has great promise as a technique for screening for cervical cancer and pre-cancer in resource-poor settings, such as India, using conventional smears stained manually by immunocytochemistry. We now require appropriately powered larger-scale studies of the suitability of this method for developing nations. In due course these studies should incorporate cost-benefit analyses.
